# SARS-CoV-2 variant Omicron: currently the most complete “escapee” from neutralization by antibodies and vaccines

**DOI:** 10.1038/s41392-022-00880-9

**Published:** 2022-01-28

**Authors:** Maochen Li, Fuxing Lou, Huahao Fan

**Affiliations:** College of Life Science and Technology, Beijing University of Chemical Technology, 100029, Beijing, China

**Keywords:** Vaccines, Infectious diseases

**Fig. 1:**
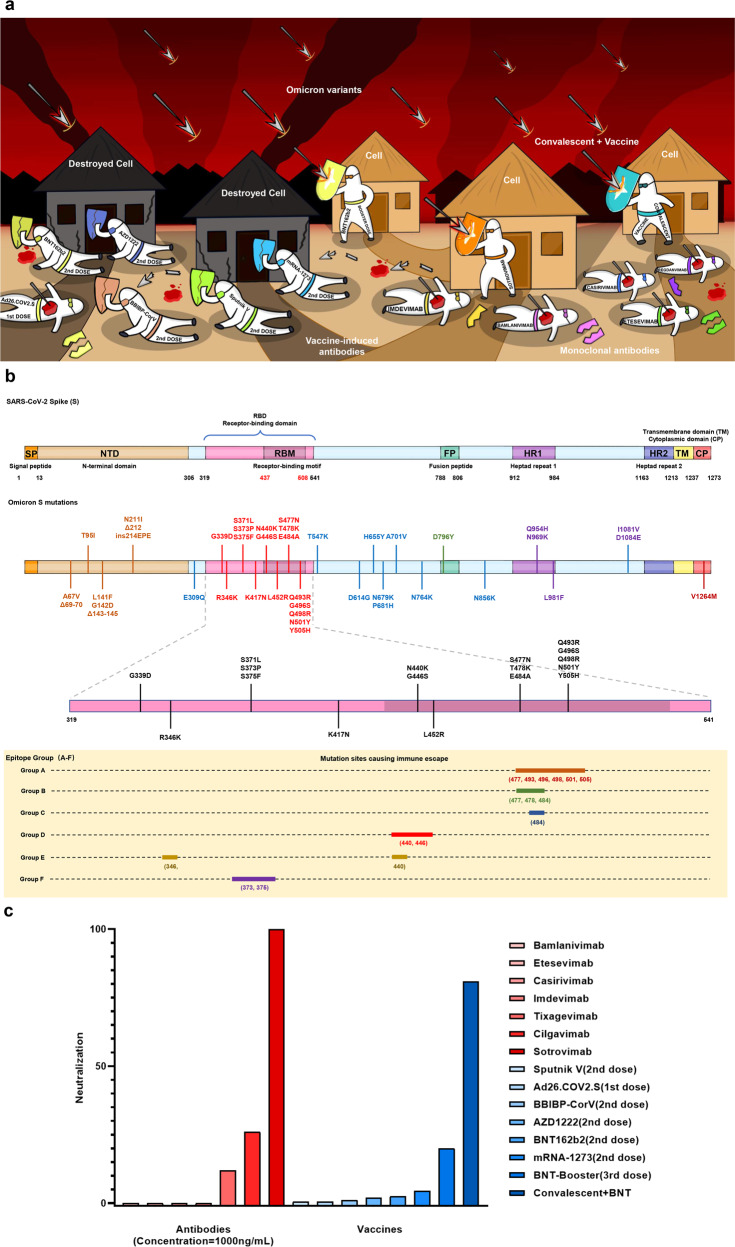
A schematic illustration of the variant Omicron escaping approved antibodies and vaccines. **a** FDA has approved several S protein-targeted monoclonal antibodies, in which Bamlanivimab, Etesevimab, Casirivimab, Imdevimab and CT-P59 (Regdanvimab) as well as the serum of all kinds of 2nd vaccine dose, fail to neutralize Omicron variant, while Sotrovimab and convalescent plus vaccinated plasma maintain the efficacy of Omicron variant. **b** Amino acid substitutions of Omicron variant in the spike protein, some of these mutations in RBD may affect the neutralization activity of group A–F antibodies, respectively. **c** The neutralizations of seven approved antibodies (Tixagevimab, Cilgavimab, Sotrovimab, Bamlanivimab, Etesevimab, Casirivimab, and Imdevimab) and six vaccines (BNT162b2, mRNA-1273, AZD1222, Sputnik V, BBIBP-CorV, Ad26.COV2.S) against Omicron are displayed, among which Bamlanivimab, Etesevimab, Casirivimab, and Imdevimab completely lose the neutralization while Sotrovimab still can neutralize Omicron variant effectively. All the 1st and 2nd dose vaccine serum fail to neutralize Omicron variant, and both the serum from convalescent patient with vaccination and booster dose of BNT162b2 retain neutralization activity against Omicron variant

On December 23, 2021, five groups published their research results of clinical-approved monoclonal antibodies, convalescent serum, and vaccine serum against B.1.1.529 (Omicron) on *Nature*.^1–5^ As a SARS-CoV-2 variant of concern (VOC), variant Omicron named by World Health Organization (WHO) with more mutations possesses the increased immune escape ability than all previous reported circulating variants, which has attracted extensive attention all over the world (Fig. 1a).

As of 29 December 2021, COVID-19 has caused at least 282.9 million infections and 5,417,752 deaths (https://coronavirus.jhu.edu/). Antibody therapies and vaccination are undoubtedly effective means to alleviate medical pressure and control the epidemic. At present, U.S. Food and Drug Administration (FDA) has approved at least seven spike protein-targeted monoclonal antibodies including Tixagevimab (COV2-2196), Cilgavimab (COV2-2130), Sotrovimab (S309), Bamlanivimab (LY-CoV555), Etesevimab (CB6), Casirivimab (REGN10933) and Imdevimab (REGN10987) for clinical use (https://www.fda.gov/). However, the emergence of Omicron variant with high infectivity and immune escape ability brought indelible challenges to the antibody therapy and vaccines. The number of Omicron sequences detected worldwide has expanded at least 10-fold in only 10 days, indicating the strong possibility of Omicron variant becoming the new dominant variant.^2,3,6^

Cao, Y et al.^1^ established a magnetic-activated cell sorting based on yeast display platform and characterized the receptor-binding domain (RBD) escaping mutation profile for 247 neutralizing antibodies (NAbs) obtained from convalescent serum and vaccine recipient serum, and these NAbs were classified into 6 epitope groups from A to F. Group A to D antibodies overlap with class 1-2 antibodies defined by Planas et al.,^2^ and group E-F antibodies are similar to class 3–4 antibodies. Group A antibodies usually cover the ACE2-binding motif and are affected by amino acid(aa) mutations of 417/420/456/475 and 455 sites. Group B antibodies target the left shoulder of RBD and are very sensitive to the aa486/487 and 476 mutations. Group C antibodies can bind to both “up” and “down” RBD with the highest neutralizing activity, while they are very sensitive to aa484 mutation. Group D antibodies depended on the ring structure formed by 440-449 residues will rotate spatially and act on S309 site. Compared with above four common groups, groups E and F antibodies are relatively rare, the typical members of them were isolated from SARS-CoV-1 convalescent, and they do not interact directly with ACE2. Group E antibodies recognize complex protein/carbohydrate structures, including N-linked glycan on N343. These antibodies will be affected by aa339, 345 and 346 mutations. Group F antibodies binding to hidden epitopes of RBD are sensitive to aa374, 376 and 378 mutations. All above six groups of antibodies could be affected by different mutations in Omicron, respectively, as shown in Fig. 1b.

The neutralization efficacy of seven FDA-approved antibodies and eight antibodies in development (Brii-196, Brii-198, VIR-7832, CT-P59, ADG-2, 910-30, DH1047, S2X259) in alone or combination use against Omicron variant were estimated.^2,3,5^ Among them, Bamlanivimab, Etesevimab, Casirivimab, Imdevimab, CT-P59 (Regdanvimab) and 910-30 retained no neutralizing activity to Omicron variant.^1–3,5^ The mutation Q493R can induce the disappearance of hydrogen bonds or the collision of antibody CDRH3 region by causing the change of amino acid spatial structure, which may explain the neutralization failure of Etesevimab (class 1/group A) and Bamlanivimab (class 2/group C). Other two mutations N440K, G446S reduce the neutralization activity of class 3/group D antibody Imdevimab by forming steric hindrance. Sotrovimab, Brii-196, Brii-198, ADG-20, DH1047, and S2X259 retained neutralizing activity against Omicron variant, and they all belong to class 3–4/group E-F except for Brii-196. However, no antiviral activity was detected in Brii-198 and DH1047 against B.1.1.529 + R346K pseudovirus, supporting that group E antibodies are sensitive to aa346 mutation.^1,3^ Surprisingly, S371L may affect partial antibodies in all classes by changing the conformation of mixed protein/carbohydrate epitope involving N343-N-linked glycan.^1,2^ Both Bamlanivimab/Etesevimab cocktail and Casirivimab/Imdevimab cocktails lost the neutralizing activity, while Tixagevimab/Cilgavimab cocktail was still effective for neutralizing Omicron variant.^3,5^ All above results indicated that currently clinically available class 1-2 antibodies were difficult to neutralize Omicron variant, and the use of class 3–4 antibodies could be under consideration for further COVID-19 patients’ treatment.

Moreover, the efficacy of vaccines was also severely affected by Omicron variant. The serum obtained from Ad26.COV2.S (single dose), Sputnik V (double dose) and BBIBP-CorV (double dose) recipients all showed negligible neutralization against Omicron variant,^5^ and the neutralizing antibodies against Omicron variant were not detected in BNT162b2 and AZD1222 vaccine recipients in the 5th month after the 2nd dose.^2^ After the booster dose, neutralizing activities against Omicron variant of all BNT162b2 vaccine serum significantly increased, but still decreased at least 4-fold compared with the efficiency against Wuhan-Hu-1 strain^2–5^ (Fig. 1c). It is worth noting that the serum neutralizing antibodies level from previous-infected recipients with booster dose is higher than naive-uninfected counterparts,^3,5^ which further suggested that positive vaccination should be encouraged whether infected by SARS-CoV-2 or not, and the booster dose should be taken in time to maintain the efficiency.

In conclusion, these recent studies evaluated the neutralization activity of currently authorized or approved antibodies and approved vaccines against Omicron variant, and provided significant guidance for the future individualized antibody therapy and mass vaccination: (1) For Omicron variant-infected patients, the treatment with group E/F antibodies should be taken as priority, and isolation measures are necessary to avoid the emergence and transmission of immune escape strains against E/F epitopes antibodies during treatment; (2) For the SARS-CoV-2 vaccine design, the sequence of seed strain should be updated according to Omicron variant, and mixed vaccination to avoid immune escape deserves clinical trials; (3) Positive vaccination is indispensable regardless of a history of SARS-COV-2 infection or not, and the booster dose should be given in a timely manner to maintain the efficiency;^6^ (4) Continuous mutations of spike protein could undermine the effect of current vaccination, while widespread vaccination in combination with the highly effective oral anti-COVID-19 drugs targeting conservative regions (e.g., 3CL, RdRp), will greatly contribute to the end of the epidemic.^7^
